# The Monster Monopoly

**Published:** 1879-09

**Authors:** 


### THE MONSTER MONOPOLY.

Mr. Daniel Walford, a workingman of New York, in a recent address, contrasted duties according to government returns paid on the enormous amount of $739,000,000 worth of liquor manufactured in one year, with the capital expended on other manufactures: viz., flour and meal, $224,000,000; cotton goods, $115,000,000; boots and shoes, $90,000,000; clothing, $70,000,000; woolen goods, $69,000,000; books, newspapers, and job printing, $42,000,000. Showing that the value of the destructive, crime, and poverty-producing article of liquor exceeds by $129,000,000 that of six of the most important and necessary articles of manufacture and industry. He also stated the significant fact that while multitudes of the useful mills and manufactories had been compelled to stop, he did not know of a single instance where a brewery or distillery had stopped manufacturing from want of trade.

### *CONSTIPATION.*

It is doubtful if consumption numbers as many victims as are stricken down by the various diseases that result from habitual constipation. True consumption is an inherited disease. It may remain always dormant, but when aroused to action, decay commences at a point circumscribed, and gradually extends—unless arrested—until so much of the lungs become involved that vital action ceases. The evils of constipation result from inattention to the calls of nature, and usually commence with children whose habits are not closely looked to by their parents. The processes of nature are always active while life lasts. When effete matter is retained a moment beyond the time its expulsion is demanded, the system commences its efforts to get rid of it. When the natural egress is checked, the absorbents carry the more fluid portions of the poisonous mass into the circulation, and it becomes diffused throughout the body. The more solid or clay like portion is forced into the lower rectum where it becomes firmly impacted, thus cutting off the circulation in the small blood vessels, causing painful engorgement known as piles and hemorrhoids. A continuance of these troubles often results in fissure, fistula, or cancer. The trouble is seldom confined here. As a result of the blood poisoning we almost invariably find more or less dyspepsia, with decided derangement of the functions of the heart, liver, and kidneys, accompanied by headache and nervous debility, often verging on paralysis.

Persons of sedentary habits are most subject to constipation and its sequences. Before the plan of treatment devised by M. Nelaton was introduced, habitual constipation was a disease of far more serious import than at the present time. All physicians admit that cathartics afford only temporary relief, and catharsis cannot be induced except by stimulating the bowels with irritants, and as this irritative process gradually weakens their peristaltic action, the relief from them steadily diminishes until disease of some vital organ sets in, and terminates life. Relief from injections is just as pernicious, with the added danger of strictures of the rectum. With these facts in view, M. Nelaton commenced his investigations, which resulted in proving beyond question that in all uncomplicated cases of constipation, the obstruction exists solely in the lower portion of the rectum, and within reach of local remedies. For the radical cure of the various diseased conditions, he invented "Nelaton's Suppositories." It is said they succeeded beyond his own expectations. But in old and severe cases their use must be persisted in until the cure is complete.

---

Nelaton's Suppositories are sold by druggists at $1 00 a box. Hall & Ruckel, wholesale druggists, New York, are agents for the United States, and send them free by mail on receipt of price.

